# The Measurement and Clinical Implications of the CA/C Ratio in Binocular Vision: A Literature Review

**DOI:** 10.22599/bioj.455

**Published:** 2026-02-11

**Authors:** Marc Argilés

**Affiliations:** 1 Universitat Politècnica de Catalunya, Spain

**Keywords:** CA/C, AC/A, vergence, accommodation, convergence

## Abstract

**Aim:**

This work summarises research on the convergence accommodation to convergence (CA/C) ratio, including the methods used to measure it in both laboratory and clinical settings. It explores the role of the CA/C ratio in the diagnosis and treatment of binocular and accommodative dysfunctions, considers its potential association with myopia, discusses age-related differences, examines its relationship with the accommodative convergence to accommodation (AC/A) ratio and reviews established normative values.

**Methods:**

A literature-based narrative review was carried out on the CA/C ratio and/or convergence accommodation, vergence accommodation and vergence interaction.

**Results:**

Research to date indicates that the CA/C plays an important physiological role in binocular vision, as well as in binocular and accommodative dysfunctions. It declines with age, does not appear to be related to the onset of myopia, and has a normative value (for adults) of 0.08 ± 0.02 D/Δ, 95% confidence interval (CI) of (0.07, 0.08).

**Conclusions:**

This literature review highlights the complexity of measuring the CA/C ratio, its potential value in visual assessment and its relevance to binocular and accommodative dysfunctions. Although it is not commonly measured in clinical practice, current evidence suggests that the CA/C ratio plays an important role in visual diagnosis and management. To support its clinical integration, future research should focus on developing standardised protocols for its evaluation.

## Introduction

Human binocular vision involves the interaction between vergence and accommodation to achieve and maintain clear and single vision. If focus needs to be oriented towards a target at a specific distance in space, there must be a synergy between vergence and accommodation to make the target appear single and clear (Benjamin, 2006; Scheiman and Wick, 2008). The horizontal vergence system involves convergence and divergence, and the accommodative system, composed of the ciliary muscle and the crystalline lens, is responsible for clarity of focus. These interactions are expressed in their respective ratios, the accommodative-convergence/accommodation (AC/A) and the convergence-accommodation/convergence (CA/C), two components that quantify the degree and manner of interaction between vergence and accommodation. The AC/A ratio represents accommodation-induced vergence (accommodation vergence), whereas the CA/C ratio refers to accommodation induced by vergence (vergence accommodation). Both ratios have an inverse relationship; usually, a high AC/A ratio comes with a lower CA/C ratio (Horwood and Riddell, 2014; Horwood, Toor and Riddell, 2014). Furthermore, the CA/C ratio shows how much accommodation is driven by a vergence stimulus (Benjamin, 2006, Chapter 5; Grosvenor, 2007, Chapter 4; Scheiman and Wick, 2008, Chapter 15).

To date, considerable research has been conducted on the CA/C ratio. However, the challenges associated with accurate measurement make it rarely utilised at the clinical level. This lack of clinical application can be attributed to several factors. The complexity and variability of measurement techniques, coupled with the absence of standardised protocols, have made the CA/C ratio less accessible for routine practice. Additionally, the specialised equipment required for accurate assessment is often not available in standard clinical settings, further contributing to its neglect. Another contributing factor may be how the roles and principles of the CA/C ratio are taught, as variability in education and training could affect clinicians’ familiarity and confidence with its application. All these factors have contributed to the CA/C ratio receiving less emphasis and clinical use than the AC/A ratio. However, the CA/C relationship may, in fact, play a more critical role in everyday visual function (Horwood, 2016). This is because convergence, driven by the desire for accurate stereopsis, tends to be more precise and arguably more functionally significant than accommodation, which operates over a broader depth of focus. This literature review provides an overview of the different methods employed to assess the CA/C ratio, outlines the diagnostic and therapeutic approaches for binocular and accommodative disorders, explores its possible link to myopia, addresses age-related variations, investigates its connection to the accommodative convergence to accommodation (AC/A) ratio and examines existing normative data.

## Methods

This manuscript is based on a narrative literature review. The PubMed, Scopus, and Web of Knowledge databases were searched using the keywords ‘CA/C ratio’, ‘convergence accommodation’, ‘vergence accommodation’ and ‘vergence interaction’ with no restrictions on the year of publication. The literature reviewed spans the years 1949–2023. Only articles written in English were included, and duplicate entries were removed. Classical textbooks in vision science were reviewed, specifically those sections explaining the CA/C ratio. The textbooks were selected on the basis of their inclusion in the recommended reading lists of core binocular vision and visual physiology courses at the Faculty of Optics and Optometry (Polytechnic University of Catalonia). These texts did not appear through the database search but were deliberately included due to their authoritative explanations of the CA/C ratio and their relevance in clinical practice. The specific textbooks reviewed were: Borish’s Clinical Refraction by Benjamin (2006), Primary Care Optometry by Grosvenor (2007), Models of the Visual System by Hung and Ciuffreda (2013) and Clinical Management of Binocular Vision by Scheiman and Wick (2008). Additional specific literature was searched to supplement the discussion sections, particularly on topics such as myopia control.

## Results

A total of 31 studies were identified from the search using the term ‘CA/C ratio’, and 45 studies were found using keywords related to convergence accommodation, vergence accommodation, accommodation convergence and vergence interaction. Additionally, four textbooks were included in the review (Benjamin, 2006; Grosvenor, 2007; Hung and Ciuffreda, 2013; Scheiman and Wick, 2008). The following section provides a detailed analysis of the literature, explaining the role of the CA/C ratio within current models of vergence and accommodation. It discusses different measurements of response and stimulus, the units of measurement, methods for opening the loop of the accommodative system to measure the CA/C ratio, and its role in visual examination, binocular and accommodative dysfunctions, myopia, vision therapy and age-related differences. Furthermore, a meta-analysis of normative values is introduced.

## Discussion

### Models in vergence and accommodation

**Figure 1:**
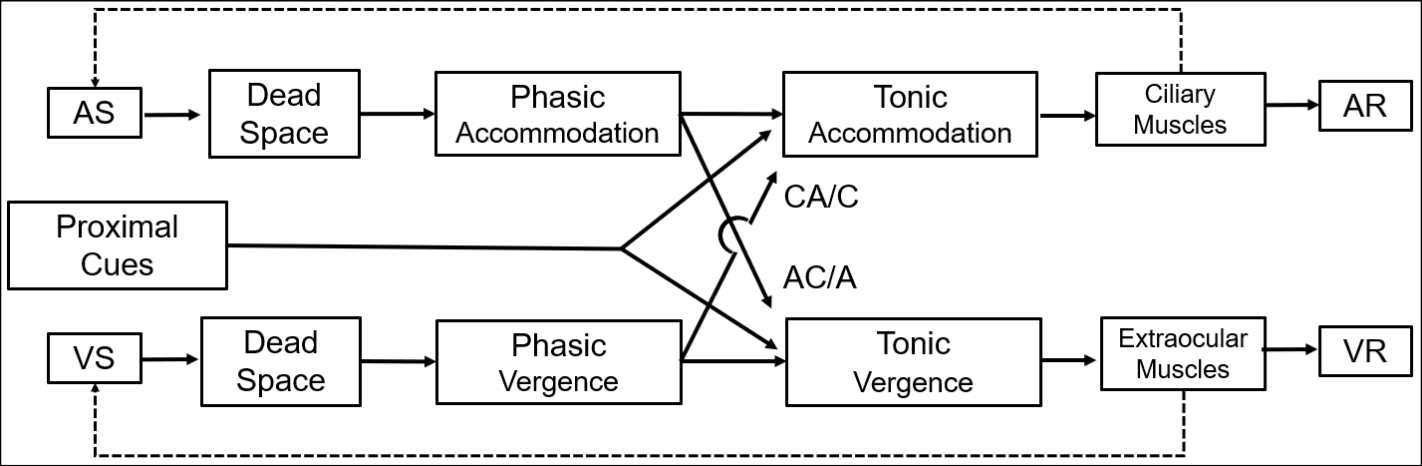
Schematic adaptation of the static dual accommodation and vergence system model. This model is based on the works of Hung, Ciuffreda and Rosenfield (1996). The Dead Space for accommodation, upper row, corresponds to the depth of focus, while vergence Dead Space, bottom row, refers to Panum’s area. The phasic and tonic accommodation for each system is represented, as well as the negative loops. The vergence controller gain (VCG) is represented as a flow arrow between the VS and VR. Proximal cues contribute to the dual system. AS = Accommodative Stimulus, VS = Vergence Stimulus, AR = Accommodative Response, VR = Vergence Response.

Previous studies have used static-duality models to explain and quantify all the components involved in the accommodative and vergence response. The term ‘static’ implies that the model focuses on a steady-state or equilibrium condition of the accommodative and vergence systems rather than analysing dynamic changes over time. The term ‘duality’ highlights that the model incorporates and quantifies the two interrelated systems of accommodation and vergence. Earlier work by Schor (Schor and Kotulak, 1986; Schor, 1988a; Schor and Horner, 1989) and Hung (Hung, 1991; Hung, 1992) led to the currently accepted model of Hung, Ciuffreda and Rosenfield (1996), which is illustrated schematically in Figure 1. This model incorporates disparity, blur and tonic inputs as well as proximal cues to contribute to the vergence and accommodative response (VR and AR), respectively. Both vergence and accommodation are subject to inaccuracy, resulting in a mismatch between stimulus and response. In the vergence system, a small misalignment of the eyes relative to the fixation target is known as fixation disparity. In the accommodation system, a similar mismatch between the response and the stimulus is referred to as an accommodative error, which may appear as a lag (an insufficient response to the stimulus) or a lead (an excessive response). This model also incorporates the crosslink between the two systems, represented in the centre of Figure 1. One such crosslink is the CA/C ratio, working in conjunction with the AC/A ratio. The phasic system refers to the rapid adjustment of vergence and accommodation, driven by cues such as retinal disparity and blur cues, respectively. The tonic component represents the baseline steady-state level of vergence and accommodation in the absence of visual stimuli.

Another crucial aspect is the presence of negative loops in these models. Under normal binocular conditions, blur and retinal disparities are present, providing the primary motor drives to their respective systems, which correspond to a ‘closed-loop’ condition. These negative feedback loops (indicated by the dashed lines in Figure 1) can be ‘opened’ by eliminating either retinal disparity (lower loop for vergence) or blur (upper loop for accommodation). This concept is important for understanding why and how the CA/C ratio is measured. The underlying physiological and mathematical models of these feedback systems help explain the dynamic interaction between accommodation and vergence, forming the foundation for CA/C measurement methodology (Eadie and Carlin, 1995; Jiang, Hung and Ciuffreda, 2002; Hung and Ciuffreda, 2013, Chapter 9).

### Units of measurement

The CA/C ratio can be expressed in either sphere dioptres (D) divided by prism dioptres (D/∆), or in sphere dioptres divided by metre angle (D/MA). The metre angle (MA) is another metric that is often referenced but is not widely used in clinical settings (Schor, 1999). MA represents the reciprocal of the fixation distance (MA = 1/distance in metres) and describes the amount of convergence or accommodation required to fixate a target at 1 m. Because the convergence demand at a given distance depends on the interpupillary distance (IPD), the MA value varies with IPD. This metric facilitates direct comparison with the AC/A ratio in terms of their relative strengths and contributions to the near-reflex complex. The ocular convergence in prism dioptres is equal to the IPD multiplied by the MA. Thus, for example, for an IPD of 6 cm and a stimulus at 0.4 m, which corresponds to an accommodative stimulus (AS) of 2.50 D, the ocular convergence or vergence stimulus is 6 × 2.50 = 15 ∆. To convert from D/∆ to D/MA, the value must be multiplied by the IPD, and to convert from D/MA to D/∆, the value must be divided by the IPD. For adults, an average IPD of 6.0 cm is commonly used, and for children, an average of 5.5 cm is a reasonable estimate (MacLachlan and Howland, 2002).

The CA/C ratio reflects the extent to which the convergence effort influences accommodation. For example, suppose that an individual views a stimulus from 0.4 m. At this distance, the total accommodation demand is 2.5 D to bring the stimulus into focus within the viewer’s depth of focus. However, this accommodation demand is not met solely by blur-driven and proximal cues. Instead, part of the response is contributed by the coupling between convergence and accommodation, which is quantified by the CA/C ratio. Now, let us assume a CA/C ratio of 0.08 D/∆. This means that for every 1 ∆ of convergence, the accommodation system provides 0.08 dioptres of accommodation (D). If a convergence demand of 15 ∆ is required to view the stimulus comfortably at 0.4 m, this induces 1.2 D of accommodation (calculated as 15 ∆ × 0.08 D/∆ = 1.2 D). In this example, the total accommodation response includes the 1.2 D driven by the convergence effort and the remaining 1.3 D from other sources, such as blur and proximal cues, to meet the total 2.5 D demand.

To further clarify the role of the CA/C ratio, we compared the following scenarios. If the CA/C ratio were 0.10 D/∆, the same 15 ∆ of convergence would induce 1.5 D of accommodation. This reduces the reliance on other (blur and proximal) cues to 1.0 D. Conversely, with a lower CA/C of 0.05 D/∆, the 15 ∆ of convergence would contribute only 0.75 D of accommodation, requiring blur and proximal cues to provide a greater share (1.75 D) to meet the demand.

### Methods to open the accommodative loop

To accurately measure the CA/C ratio, the accommodative system must be placed in an ‘open-loop’ condition, which removes the influence of blur-driven accommodation. This ensures that only the vergence stimulus is present, allowing the contribution of convergence to be assessed independently of accommodation. In other words, assessing the CA/C ratio requires measuring the accommodative response in a controlled environment in which blur and proximal cues are absent. By removing these blur-driven inputs, the measurement focuses solely on the relationship between convergence and accommodation, ensuring that the derived CA/C ratio reflects the true interaction between these two systems without interference from other accommodative triggers.

There are two main ways to perform this. First, a binocular pinhole can be used. Under this condition, the depth of focus is increased considerably (see Figure 1), opening the accommodative loop (Jiang, Hung and Ciuffreda, 2002), with a few studies using this technique (McLin Jr, Schor and Kruger, 1988; Kotulak, Morse and Rabin, 1995). However, maintaining fixation through the pinholes while simultaneously changing vergence can be quite challenging.

**Figure 2:**
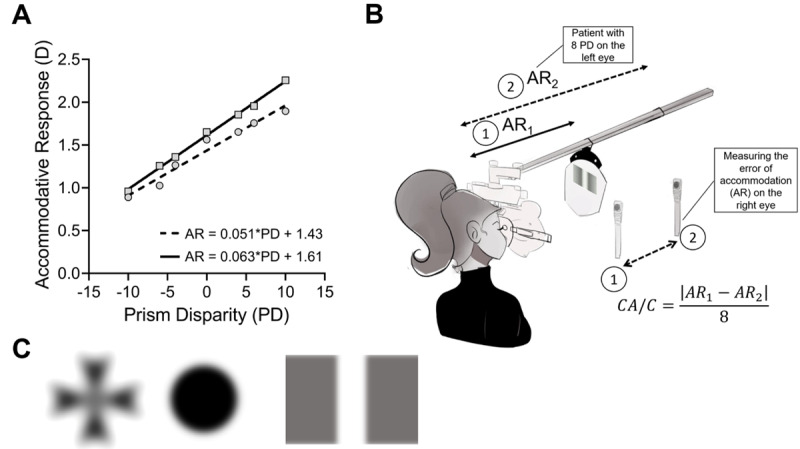
Panel **A.** Example of two patients deriving the CA/C ratio from the slope between accommodative response (AR) and prism disparity (PD, Δ), which represents the vergence stimulus (VS), using linear regression (from the equation y = mx + b), where ‘m’ is the slope. Note that the ‘dashed line’ patient has a CA/C ratio of 0.051 D/Δ, while the other patient has a CA/C ratio of 0.063 D/Δ. Some studies, instead of using prism disparity, have utilised the metre angle (MA) for the ‘x’ axis and subsequently derived the CA/C ratio slope in units of D/MA. Panel **B.** Schematic representation of measuring the stimulus gradient CA/C ratio using the NOTT technique and a DOG image. The formula to calculate the CA/C gradient ratio is shown in the bottom-right of the image. Panel **C.** Examples of DOG images created using Gaussian Blur (σ = 30) with the software Image J. From left to right: Black Maltese Cross, Black Circle, and a 0.1 cpd vertical stripe on a grey background. The last image closely resembles the reverse side of the Wesson fixation disparity card (Bernell, Mishawaka, USA).

The second method involves using a Difference of Gaussians (DOG) stimulus. Research has shown that low spatial frequencies, approximately 0.2 cycles per degree (cpd), are particularly effective in this context. These low spatial frequencies create an accommodative response closely related to viewing an empty field (Charman and Tucker, 1977). Following a Kernel of Gaussian, which is a mathematical matrix derived from a Gaussian function and widely used in signal processing, computer vision and machine learning (Tsuetaki and Schor, 1987), blur does not induce a cue to accommodation, which then opens the loop of the accommodative system (Tsuetaki and Schor, 1987; Kotulak, Morse and Rabin, 1995; Scheiman and Wick, 2008, Chapter 15). Some DOG stimuli have been used by some authors with the free available software Image J (National Institutes of Health, USA), using a Gaussian blur filter with Sigma = 16 (Sweeney *et al*., 2014). Moreover, the reverse side of the Wesson fixation disparity card (Bernell, Mishawaka, USA) has a DOG image. Examples of DOGs using a Gaussian blur filter are shown in Figure 2 (part C). Most studies use different versions of a DOG stimulus to measure the CA/C (Daum *et al*., 1989; Nonaka, Hasebe and Ohtsuki, 2004; Brautaset and Jennings, 2006; Fukushima *et al*., 2009; Vedamurthy *et al*., 2009; Nilsson and Brautaset, 2011; Simmons and Firth, 2014; Sweeney *et al*., 2014; Babinsky, Sreenivasan and Candy, 2015; Neveu *et al*., 2015; Neveu *et al*., 2016), which can be printed or projected on a monitor or by using an LED with a diffusor (Nonaka, Hasebe and Ohtsuki, 2004; Simmons and Firth, 2014). Interestingly, a recent study showed that a defocus flicker of different chromatic stimuli deactivates accommodation (Rodriguez-Lopez, Hernandez-Poyatos and Dorronsoro, 2023), which can be potentially used to evaluate the CA/C ratio.

### Measurements of response and stimulus

There is a distinction between stimulus and response. For instance, it is typically assumed that a –2.00 D lens will prompt an accommodative response of 2.00 D, or that viewing a target at 40 cm with an IPD of 6 cm will result in a convergence of 15∆. However, these assumptions may not accurately represent the actual responses in real-life scenarios. In order to determine the actual accommodation response, we need specific instruments, (i.e., optometers, Power Refractors) (Kotulak, Morse and Rabin, 1995; Allen and O’Leary, 2006; Brautaset and Jennings, 2006; Nilsson and Brautaset, 2011; Horwood and Riddell, 2013a; Simmons and Firth, 2014; Neveu *et al*., 2016; Doyle, Saunders and Little, 2017), and for the vergence response, eye-trackers (Rovira-Gay *et al*., 2023). Some investigations have shown that CA/C ratio stimulus and response have a relationship between them (Tsuetaki and Schor, 1987; Wick and Currie, 1991; Sweeney *et al*., 2014; Neveu *et al*., 2015).

In research, it is mandatory to know the exact response of both accommodative and vergence systems. The measurement of the accommodative responses to determine the CA/C ratio used Power Refractors or optometers in most studies (Kotulak, Morse and Rabin, 1995; Bobier *et al*., 2000; Nonaka, Hasebe and Ohtsuki, 2004; Brautaset and Jennings, 2006; Fukushima *et al*., 2009; Vedamurthy *et al*., 2009; Nilsson and Brautaset, 2011; Horwood and Riddell, 2012a; Horwood, Toor and Riddell, 2014; Simmons and Firth, 2014; Sweeney *et al*., 2014; Babinsky, Sreenivasan and Candy, 2015; Neveu *et al*., 2016). The CA/C values in the studies measuring AR were mostly calculated from the slope between AR and VS. The slope between the accommodative response to different vergence stimuli is the CA/C ratio (Figure 2, part A, for example). It is important to keep in mind that most eye care professionals require a practical method to evaluate the CA/C ratio, as advanced instruments like Power Refractors or optometers are rarely available in typical clinical settings. Therefore, there is a clear need to identify accessible and reliable clinical techniques for estimating the CA/C ratio.

The CA/C ratio is also measured with the gradient technique, which evaluates the difference between two accommodative responses induced by a prism (a similar technique to the use of sphere dioptres for the evaluation of AC/A ratio stimulus). The CA/C gradient ratio is measured using the NOTT method, which employs a phoropter and a retinoscope to assess accommodation (Tsuetaki and Schor, 1987; Daum *et al*., 1989). Some authors state that the light of a retinoscope does not induce a cue in the accommodative system (Tsuetaki and Schor, 1987), but only one investigation has used a Difference of Gaussians (DOG) with a hole in the centre using the NOTT technique (Goss *et al*., 2015). Proximal cues are kept constant during this method, although they influence the measure in the response method (Neveu *et al*., 2015). Using this technique, the error made from not being at the right angle with the retinoscope is compensated, because both errors of accommodation are measured in the same way (Benjamin, 2006, Chapter 21). By using the NOTT method, an eye care professional can measure the accommodative response in the right eye with (AR2) and without a prism (AR1), placing the prism over the left eye to stimulate binocular vergence (Figure 2, part B). The use of binocular cross-cylinder (BCC) to calculate the error of accommodation is another technique derived using the NOTT technique (Goss *et al*., 2015). Additionally, graphical analysis has been extensively studied to help the diagnosis of binocular and accommodative dysfunctions, but only one study included a method to calculate the CA/C ratio using this analysis (Schor and Narayan, 1982). A schematic representation of the clinical measurement of the gradient CA/C ratio stimulus is presented in Figure 2, part B.

A few studies have used the gradient method to evaluate the CA/C ratio (Schor and Narayan, 1982; Tsuetaki and Schor, 1987; Daum *et al*., 1989; Brautaset and Jennings, 2006; Neveu *et al*., 2015). The prism of choice is 8 Δ base-out (BO) in most studies (Tsuetaki and Schor, 1987; Brautaset and Jennings, 2006; Neveu *et al*., 2015), although other authors used 8 Δ base-in (BI) (Schor and Narayan, 1982), or 6 Δ BI (Daum *et al*., 1989). Nevertheless, it seems that the amount of prism selected does not influence the final measurement in the gradient CA/C ratio method (Hirani and Firth, 2009; Simmons and Firth, 2014). In addition to the magnitude of the prism, the duration for which the prism remains in front of the eyes is unclear. Since tonic vergence (Schor, 1992) and vergence adaptation (Schor and Narayan, 1982; Schor, 1988b; Schor and Horner, 1989; Polak and Jones, 1990; Schor, 1999) influence the measure of the CA/C, some authors have opted for leaving the prism for less than 30 seconds to prevent vergence adaptation (Sweeney *et al*., 2014), whereas others used 5 minutes for vergence adaptation before the measurement of CA/C (Neveu *et al*., 2015).

### Diagnostic testing

Understanding vergence and accommodation models, as well as the role of the CA/C ratio, is essential for grasping the foundations of diagnostic tests in visual exploration. The fusional vergence range represents the limits of how much convergence (positive vergence) or divergence (negative vergence) an individual can use to maintain single binocular vision. During this test, vergence accommodation plays an important role, as it indicates the extent to which accommodation is driven by convergence or divergence. For example, during positive fusional vergence testing with either a prism bar or Risley prisms method, the vergence stimulus is increased while the accommodative demand remains unchanged. Then, as the base-out (BO) demand increases, the stimulation of vergence accommodation and the corresponding magnitude of the blur point are influenced by the CA/C ratio. However, although the CA/C ratio may affect the occurrence of blur, subjective perceptions of blur may not always align with measurements. In general, the higher the CA/C ratio, the greater the likelihood of reporting a blur point, as more accommodation is induced, which moves the blur outside the depth of focus (Benjamin, 2006, Chapter 21, p. 946). Another source of blur occurs during fusional vergence testing when the vergence demand exceeds the individual’s relative fusional vergence capacity. Although accommodation is generally stable within the depth of focus, increasing BI demand can reduce convergence-driven accommodation passively. When this reduction exceeds the accommodative system’s ability to compensate, perceptual blur may occur, indicating that the fusional vergence limit has been reached.

During BI prism testing, as the demand for divergence increases, the patient must relax convergence-driven accommodation. At the same time, accommodation may still be required to maintain clarity. At some point, once the fusional divergence limit is reached, the patient may begin to relax accommodation to induce additional divergence through the AC/A linkage. However, note that accommodation cannot relax beyond zero; therefore, this relaxation is only possible if the prescription is under-plussed or over-minused. On distance BI vergence testing, patients typically do not perceive a blur unless their prescription is over-minused or under-plussed. In such cases, accommodation provides additional room for relaxation and allows the patient to gain more divergence. In addition, it is important to consider pupil miosis during these measurements, as it may mask blur during fusional vergence range testing by increasing the depth of focus (Wang and Ciuffreda, 2006; Lara *et al*., 2014; Dhallu *et al*., 2019).

Moreover, during fixation disparity testing, specifically through fixation disparity curves, four types of curves have been classically defined based on Ogle’s classification (Ogle, Mussey and Prangen, 1949): Type I (considered a normal response with a symmetrically sigmoid shape between BI and BO demands), Type II (associated with an asymmetric sigmoid curve with flat segments on the BO vergence demands and a normal response with BI), Type III (associated with flat segments in the BI vergence demands and a normal response in the BO) and Type IV (little change with either BO or BI). The shape and slope of these curves provide insights into the stability of binocular vision. Variability in curve type or steepness may reflect unstable binocularity, which can result from abnormal CA/C interactions affecting how the visual system adapts to vergence and accommodative demands (Carter, 1957).

### Binocular and accommodative dysfunctions

In binocular and accommodative dysfunctions, an imbalance in ocular accommodation adaptation can lead to opposite extremes of the AC/A and CA/C ratios (Schor, 1988a), typically presenting as a high CA/C and low AC/A ratio (Benjamin, 2006, Chapter 5). These altered ratios are not solely the result of intrinsic dysfunctions; they can also be influenced by external factors such as elevated heterophoria and increased interpupillary distance (IPD). For instance, significant heterophoria places additional demands on the vergence system, requiring habitual compensation through the CA/C linkage. This can cause one system to overcompensate, disrupting the balance between the two ratios. Likewise, a high IPD increases the vergence demand, particularly during near tasks. Together, these imbalances can contribute to visual symptoms during activities that require sustained near vision.

The CA/C relationship depends on mechanisms such as slow vergence and accommodative adaptation (Schor, 1988b; Schor and Horner, 1989). For example, in individuals with convergence insufficiency, the balance between accommodation and vergence is disrupted. A high CA/C ratio means that even a small amount of convergence effort can trigger an excessively large accommodative response, theoretically resulting in blurring and visual discomfort (Scheiman and Wick, 2008).

On the other hand, a low AC/A ratio reflects a weak convergence response for a given amount of accommodation. This can result in under convergence during near tasks, leading to symptoms such as double vision, eye strain and difficulty in sustaining focus. As a result, individuals with convergence insufficiency often exhibit a greater exophoric deviation at near and reduced accommodative amplitude (Scheiman and Wick, 2008).

Nonaka and colleagues observed no significant difference in the CA/C ratio between control participants and those with intermittent exotropia or decompensated exophoria; however, they reported a significant difference in the AC/A ratio. The mean CA/C ratio was 0.080 ± 0.043 D/Δ or 0.48 ± 0.26 D/MA (Nonaka, Hasebe and Ohtsuki, 2004). Another investigation found significant differences between patients with normal binocular function and excess of convergence, mean 0.10 ± 0.02 D/Δ and 0.07 ± 0.03 D/Δ, respectively (Nilsson and Brautaset, 2011). Daum *et al*. (1989) compared the CA/C ratio in patients with binocular dysfunction with and without symptoms and found no significant differences in the CA/C ratio, mean 0.06 ± 0.05 D/Δ in the control group and 0.07 ± 0.06 D/Δ in the visual symptoms group (Daum *et al*., 1989). Horwood and Riddell (2012b) evaluated the CA/C ratio in participants with intermittent distance exotropia compared with control participants in a young population and found significant differences between them, mean 1.46 ± 1.16 D/MA in intermittent exotropia, and 0.87 ± 0.29 D/MA in control (Horwood and Riddell, 2012b). This elevated CA/C ratio may explain the esophoric or even esotropic state of exotropic patients after ocular surgery. In a small subset of cases where the linkage between convergence and accommodation is particularly strong, reducing the strabismic angle surgically removes much of the need to converge. This reduction can lead to postoperative hypo-accommodation because these patients rely on convergence to drive their accommodative response.

A series of investigations conducted by Horwood and Riddell (Horwood and Riddell, 2008; Horwood and Riddell, 2013a; Horwood and Riddell, 2013c; Horwood and Riddell, 2014; Horwood, Toor and Riddell, 2014) studied how independent cues of blur and disparity reflect the magnitude or gain of AC/A and CA/C ratios. In summary, the authors concluded that the CA/C ratio is as important as the AC/A ratio and that disparity is the primary cue for both the vergence and accommodation systems, as it is more accurate than accommodation, particularly in infants, where the depth of focus is wider. Low CA/C ratios are due to a greater convergence than accommodation response, and high CA/C ratios result from a greater accommodative response than converge. These authors’ investigations help explain how different weighted cues reflect the response of CA/C and AC/A linkage (Horwood and Riddell, 2008; Horwood and Riddell, 2013a; Horwood and Riddell, 2013b; Horwood and Riddell, 2014).

### Vision therapy

Vision therapy is a sequence of neurosensory and neuromuscular activities individually prescribed to develop, rehabilitate and enhance visual skills and processing. The use of lenses, prisms, filters, occluders, specialised instruments and computer programmes is an integral part of vision therapy (Scheiman and Wick, 2008). To date, the evidence of vision therapy effect on the CA/C ratio is generally weak. Previous authors have theoretically indicated that vision therapy can help balance the relationship between vergence and accommodation (Schor and Kotulak, 1986). Hung, Ciuffreda and Semmlow (1986) studied whether specific accommodative, vergence, and accommodative and vergence training can alter the CA/C ratio, and did not find any statistical changes after training in adults (Hung, Ciuffreda and Semmlow, 1986). Brautaset and Jennings (2006) evaluated the CA/C ratio using a Power Refractor, DOG at 0.4 m, and calculated the CA/C by the gradient method using 8 ∆ BO. Ten participants performed 10 minutes of vision therapy twice daily for 12 weeks, starting with vergence jump and pencil-to-nose exercises for 2 weeks, followed by prism and spherical flipper training for 2 weeks at near (40 cm). From weeks 5–12, all four exercises were combined in two daily sessions without supervision, targeting both near and distance vergence control. After 12 weeks, they did not find a significant change in the CA/C ratio, which went from 0.142 ± 0.019 D/Δ pre-training to 0.136 ± 0.012 D/Δ post-training (Brautaset and Jennings, 2006). Thiagarajan, Lakshminarayanan and Bobier (2010) also investigated the possible change in the CA/C ratio after two weeks of vision therapy in 11 adults. In this case, the training involved progressively increasing convergence demands using BO prisms while keeping accommodation constant, conducted over multiple 25–30 minute sessions totaling 180 minutes. Positive fusional vergence range was trained using variable tranaglyphs and an aperture rule. No statistically significant differences were observed in the CA/C ratio between the pre-training and post-training assessments (Thiagarajan, Lakshminarayanan and Bobier, 2010).

Modern techniques and instruments, such as the use of virtual reality, are becoming popular in vision therapy, and one study has shown that virtual reality exposure induces changes in the CA/C ratio (Eadie *et al*., 2000). Moreover, classical instruments used in vision therapy, such as the stereoscopic mirror, which is composed of a haploscopic setup, can also induce changes in the CA/C ratio (Neveu *et al*., 2016). This occurs because stereoscopic viewing, including that used in virtual reality environments, alters the normal coupling between convergence and accommodation. Specifically, the artificial presentation of binocular disparity without corresponding blur cues leads to a mismatch between vergence and accommodation demands (Elias, Batumalai and Azmi, 2019).

The convergence at near can be increased by using mirrors that increase the pupillary distance. This disproportionate near convergence, in turn, triggers accommodation via the CA/C ratio because it increases the required change in convergence per unit in accommodation. In addition, the use of BO or BI prisms has a different impact on the change in CA/C (Miles, Judge and Optican, 1987). Thus, the CA/C as well as the AC/A, are not fixed and are adaptable to environmental factors. Scheiman and Wick (2008) reported that patients with high CA/C ratios respond better to vision therapy techniques incorporating high spherical lenses and a moderate amount of prism training. However, no studies have supported this claim. More research is needed to elucidate the plasticity of the CA/C ratio in patients undergoing vision therapy.

### Myopia

The theoretical basis for investigating the potential role of the CA/C in the development and progression of myopia stems from the visual demands of prolonged near-work activities. Sustained near tasks often result in excessive accommodative effort, which in turn may lead to transient increases in myopia, referred to as near work-induced transient myopia (NITM) (Ehrlich, 1987; Ciuffreda and Lee, 2002; Arunthavaraja, Vasudevan, and Ciuffreda, 2010; Lin *et al*., 2013) (see for a review Ong and Ciuffreda, 1995; Ciuffreda and Vasudevan, 2008; Kaur, Gurnani and Kannusamy, 2020).

However, research on the amount of CA/C ratio compared with late-onset myopia, myopia, and control participants did not find significant differences between them (Rosenfield and Gilmartin, 1988; Jiang, 1995; Allen and O’Leary, 2006), in contrast to studies that found high AC/A ratios in participants with myopia (Rosenfield and Gilmartin, 1987; Mutti *et al*., 2000; Gwiazda, Thorn, and Held, 2005; Mutti *et al*., 2017; Zhou *et al*., 2021), although other authors did not find differences in the AC/A (Chen *et al*. 2003; Chen *et al*., 2020). While current evidence does not show significant differences in the CA/C ratio between individuals with myopia and controls, the limited number of studies and variability in study designs suggest that further research is warranted to fully understand the potential role of CA/C in the onset and progression of myopia.

### Age differences

The CA/C ratio is influenced by changes in the crystalline lens over time. As the lens stiffens during presbyopia and even earlier in life, the accommodative response diminishes, leading to a lower CA/C ratio. This occurs because while accommodation decreases significantly with age due to reduced lens flexibility, convergence remains relatively stable, thereby disrupting the balance between the two systems. These changes have been well documented in studies exploring the biomechanical properties of the lens (Glasser and Campbell, 1998). Most research has shown that CA/C ratio declines with age (Bruce, Atchison and Bhoola, 1995; Rosenfield, Ciuffreda and Chen, 1995; Heron, Charman and Schor, 2001; Babinsky, Sreenivasan and Candy, 2015). Rosenfield, Ciuffreda and Chen (1995) found a decrease rate of 0.006 D/Δ by year in 42 participants with a mean age of 39.7 ± 1.97 years. Although most studies indicate the same result, the slope of regression (i.e., the decrease rate of the CA/C by year) differs from one study to another, from 0.003 D/Δ (Bruce, Atchison and Bhoola, 1995) to 0.006 D/Δ (Rosenfield, Ciuffreda and Chen, 1995) or 0.004 D/Δ (Heron, Charman and Schor, 2001).

Babinsky, Sreenivasan and Candy (2015) studied the differences between the CA/C in 50 children (2–7 years) and 13 adults (20–35 years) and found a significant difference between groups; children = 0.18 D/Δ or 0.86 D/MA, and adults 0.10 D/Δ or 0.57 D/MA (Babinsky, Sreenivasan and Candy, 2015). In addition, another study compared the CA/C between infants (n = 8) and adults (n = 6) and found significant differences between them, 0.17 D/Δ or 0.73 D/MA, and 0.042 D/Δ or 0.25 D/MA, respectively (Bobier *et al*., 2000). Moreover, near correction for presbyopic population does not change the CA/C ratio (Vedamurthy *et al*., 2009). Hence, younger people tend to have higher CA/C ratios that decline with age.

### Relationship with the AC/A ratio

The CA/C and AC/A ratios are key interconnections in the vergence and accommodation systems (see Figure 1), and although they are related, there is not a reciprocal relationship between them (Bruce, Atchison and Bhoola, 1995; Rosenfield, Ciuffreda and Chen, 1995). However, some authors have reported an inverse relationship between the two systems. Specifically, when the accommodative system adapts more than the vergence system, the AC/A ratio tends to decrease temporarily, while the CA/C ratio increases (Schor, 1988a; Schor, 1999). This is corroborated by some recent investigations (Sweeney *et al*., 2014), but not found in other previous studies (Nonaka, Hasebe and Ohtsuki, 2004). Another study found that gradient stimulus AC/A is correlated with response CA/C (Horwood and Riddell, 2013c).

Hence, the CA/C and AC/A ratios reflect how vergence and accommodation are controlled. Therefore, measuring the CA/C ratio during the clinical evaluation of binocular and accommodative dysfunctions is crucial for obtaining a more comprehensive understanding of these systems.

### Normative values

There is a significant disparity in the reported normative values of the CA/C ratio. According to most of the literature, the typical CA/C ratio is 0.5 D/MA or 0.083 D/Δ (Schor and Kotulak, 1986; Benjamin, 2006, Chapter 5; Scheiman and Wick, 2008, Chapter 15). Other authors found 0.37 D/MA or 0.061 D/Δ with the model simulations of Hung, Ciuffreda and Rosenfield (1996), and ranging from 0.060 D/Δ (Fincham and Walton, 1957), to 0.10 to 0.15 D/Δ (Fincham and Walton, 1957; Kersten and Legge, 1983; Miles, Judge and Optican, 1987), from earlier works. This high variability is probably caused by the intricate method to evaluate the CA/C, and the lower values that result. In addition, the CA/C declines with age (Bruce, Atchison and Bhoola, 1995; Rosenfield, Ciuffreda and Chen, 1995), thus differing in adults from children (Bobier *et al*., 2000; Babinsky, Sreenivasan and Candy, 2015).

Although it seems that ethnicity does not influence the magnitude of CA/C (Vedamurthy *et al*., 2012), neurodivergent populations, such as individuals with Down syndrome, exhibit reduced CA/C ratios, reflecting an altered interaction between accommodation and vergence in which retinal disparity predominates and vergence is prioritised over accurate accommodation (Doyle, Saunders and Little, 2017). Moreover, binocular, and accommodative dysfunctions such as convergence insufficiency, intermittent exotropia, convergence excess and esotropias usually have abnormal levels of CA/C (Nonaka, Hasebe and Ohtsuki, 2004; Nilsson and Brautaset, 2011; Horwood and Riddell, 2012a; Horwood and Riddell, 2012b).

Given the variability of the CA/C ratio between investigations, and in order to have a framework for normative values, we analysed the weighted mean of 11 studies that provided the mean and standard deviation (SD) of the CA/C in healthy adults without any binocular or accommodative dysfunction. Some studies and textbooks were excluded from the analysis for not providing the mean and standard deviation for the CA/C ratio (Fincham and Walton, 1957; Benjamin, 2006; Grosvenor, 2007; Scheiman and Wick, 2008). Table 1 summarises the results for the 11 studies.

**Table 1:** Summary of sample characteristics, including mean age and standard deviation (±), age range, measurement methods and CA/C ratio values (in D/Δ), presented as means and standard deviations for the studies analysed.


STUDY	SAMPLE	MEAN AGE	AGE RANGE	METHOD OF MEASUREMENT	CA/C

Neveu *et al*. (2016)	12	25.5 ± 5.00	24–36	PowerRef II DOG Slope AR/VS	0.0780 ± 0.0360

Neveu *et al*. (2015)	18	27.5 ± 4.50	21–36	Dynamic retinoscopy DOG 8 Δ BO	0.0750 ± 0.0110

Simmons and Firth (2014)	18	20.60 ± 3.22	18–31	Autorefractor DOG Slope AR/VS	0.1018 ± 0.0490

Sweeney *et al*. (2014)	25	21.00 ± 1.90		Power refractor DOG Slope AR/VS	0.0910 ± 0.0460

Nilsson and Brautaset (2011)	20	24.92 ± 3.65		Power refractor DOG 8 Δ BO	0.1000 ± 0.0200

Fukushima *et al*. (2009)	8	22.80 ± 0.90	22–24	PR-1000 DOG Slope AR/VS	0.0910 ± 0.0400

Allen and O’Leary (2006)	64	20.14 ± 1.55	18–22	Power refractor Pinholes Slope AR/VS	0.0640 ± 0.0350

Kotulak, Morse and Rabin (1995)	16	25.40 ± 3.00		Optometer Artificial pupil Slope AR/VS	0.0980 ± 0.0830

Daum *et al*. (1989)	78	25.90 ± 3.20	22–29	DOG 6 Δ BI	0.0600 ± 0.0500

Hung, Ciuffreda and Semmlow (1986)	22		18–24	Dynamic Binocular Stimulator	0.1230 ± 0.0100

Kersten and Legge (1983)	5		20–30	Optometer	0.1500 ± 0.0131


**Figure 3:**
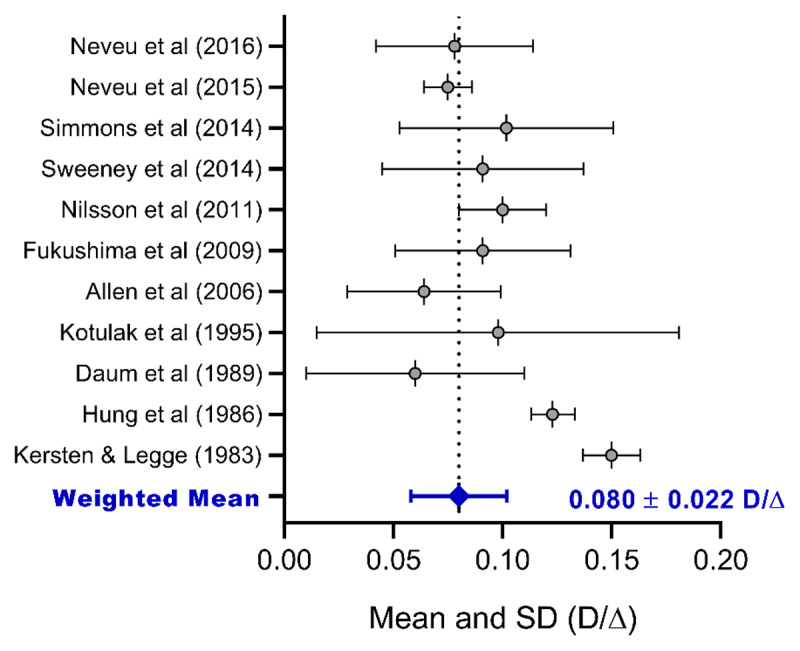
Forest plot showing the mean and SD of studies measuring CA/C in adults. The results of the studies reported in D/MA were converted to D/Δ by dividing by 6.0. The weighted mean and pooled SD are shown in the lower panel.

The weighted mean accounted for the sample population in each study. Age was provided for each study as the mean and standard deviation, the age range, or both. SPSS version 28 for Windows (Armonk, NY: IBM Corp. IBM) was used for the calculation. Figure 3 displays the results from 11 studies (Kersten and Legge, 1983; Hung, Ciuffreda and Semmlow, 1986; Daum *et al*., 1989; Kotulak, Morse and Rabin, 1995; Allen and O’Leary, 2006; Fukushima *et al*., 2009; Nilsson and Brautaset, 2011; Simmons and Firth, 2014; Sweeney *et al*., 2014; Neveu *et al*., 2015; Neveu *et al*., 2016) with a total sample of 286 adult participants, showing a weighted mean and SD of 0.080 ± 0.022 D/Δ, with a 95% confidence interval (CI) of (0.077, 0.082), or mean and SD of 0.48 ± 0.13 D/MA, which are very similar to the normative values of textbooks (Grosvenor, 2007, Chapter 5; Scheiman and Wick, 2008, Chapter 15) and other investigations conducted (Fukushima *et al*., 2009; Sweeney *et al*., 2014; Neveu *et al*., 2016). It is worth noting that the standard deviations were large in some studies, thereby demonstrating the high variability of measuring the CA/C, which is presumably due to the methodological differences and inter-subject variability.

In contrast, a few studies evaluated the CA/C ratio in binocular and accommodative dysfunctions. Nonaka *et al*. (2004) found a mean and SD of 0.48 ± 0.26 D/MA or 0.080 ± 0.043 D/Δ in younger participants with intermittent exotropia and decompensated exophoria (Nonaka, Hasebe and Ohtsuki, 2004). Brautaset and Jennings (2006) found 0.142 ± 0.019 D/Δ in adult participants with convergence insufficiency. Nilsson and Brautaset (2011) found 0.07 ± 0.03 D/Δ in participants with excess of convergence, compared to 0.10 ± 0.02 D/Δ in control subjects. Horwood *et al*. (2012b) found in intermittent exotropia 1.46 ± 1.16 D/MA, in contrast to participants without binocular and accommodative dysfunctions, 0.87 ± 0.29 D/MA (Horwood and Riddell, 2012b).

## Future Work

Although it is clear that CA/C plays an important role in the diagnosis of binocular and accommodative dysfunction, several methods can be used to measure it. The first step for future directions will be to establish a normative method, available in clinical settings, to measure the CA/C in the same way as other widespread visual diagnostic tests (i.e., AC/A), and it will need to be highly correlated with the response CA/C using the same method. Secondly, additional research is needed to explore potential changes in the CA/C resulting from vision therapy techniques. While existing studies have not identified significant differences in CA/C between myopic and non-myopic individuals, additional research is needed to clarify whether CA/C contributes to mechanisms underlying myopia development. Additionally, no studies have directly examined how CA/C magnitude is affected in patients with accommodative dysfunctions.

## Conclusions

This review of the literature highlights the intricate aspects of measuring the CA/C ratio, its potential significance in visual evaluation and its role in binocular and accommodative dysfunctions. Understanding the CA/C ratio’s role in binocular vision is crucial for the effective management of patients with or without binocular and accommodative dysfunctions. However, future studies are required to provide a standardised framework to evaluate the CA/C ratio in clinical settings and elucidate the potential role in myopia progression.
